# Venous external support enhanced coronary artery bypass grafting: a multicentric cohort experience

**DOI:** 10.1186/s13019-025-03670-w

**Published:** 2025-11-24

**Authors:** Luca Paolo Weltert, Sigrid Sandner, Paolo Centofanti, Samuel Fusca, Marija Pljakova, Vittoria Lodo, Viviana Sebastiano, Ruggero De Paulis

**Affiliations:** 1 Department of Cardiac Surgery, European Hospital, Rome, Italy; 2 UniCamillus University, Rome, Italy; 3 Department of Cardiac Surgery, Medical University Vienna, Vienna, Austria; 4 Cardiac Surgery Division, Mauriziano Hospital, Turin, Italy; 5 Department of Cardiovascular Sciences, Cardiac Surgery Unit, European Hospital, Via Ponuense. 700, 00149 Rome, Italy

**Keywords:** Coronary artery bypass grafts (CABG), Coronary artery disease, Coronary heart disease in adults, External stenting, Vein grafts

## Abstract

**Objectives:**

This multicentric study evaluated short- and mid-term clinical outcomes, with specific focus on the need for repeat revascularization, in patients undergoing coronary artery bypass grafting (CABG) with VEST supported saphenous vein graft (SVG).

**Methods:**

A total of 397 patients underwent CABG in a three-center setting, with or without concomitant procedures, with at least one SVG supported with an external stent. Open vein harvesting was performed in 80.3% of patients. The majority (73.3%) of patients underwent on-pump CABG, 6.8% of patients had concomitant valve surgery, and the average number of grafts per patient was three. Patients were followed for major adverse cardiac and cerebral events (MACCE) for a median duration of 24 (1-101) months.

**Results:**

Overall, 469 of 654 SVG (71.7%) received external stents. Freedom from MACCE at 1, 3, and 5 years was 95.1% (SE 0.011), 85.4% (SE 0.021), and 82.4% (SE 0.030) respectively. Revascularization rates in territories which were grafted with a stented SV was low in general (1.28%) and statistically significantly lower than in territories grafted with a non-stented SVG (4.32%, p = 0.015). Arterially grafted territories confirmed low revascularization rates as well (0.9%).

**Conclusions:**

VEST-enhanced CABG is feasible and associated with low MACCE in real world routine practice which includes on and off pump CABG, sequential grafting, and concomitant surgery. Short- to mid-term clinical follow up suggests that VEST enhanced CABG is associated with very low target vessel revascularization rates, with most re-revascularization happening at non-grafted or non-VEST-enhanced grafted territories.

## Background

Consistent evidence has established that surgical revascularization is the preferred treatment for complex coronary disease [1]. While the left internal thoracic artery is the preferred conduit to revascularize the left anterior descending (LAD) artery, in contemporary CABG SVG is the most utilized second conduit, most likely due to its ease of use and abundance [2]. In the short term, up to 1 year post implantation, SVG performance is influenced primarily by possible kinks and twists, harvesting technique, preservation method, and the coronary target quality [3, 4]. In the long term, the development of intimal hyperplasia and accelerated atherosclerosis limit the longevity of SVG [4, 5] and 10 years post CABG, the majority of SVG are either occluded or severely diseased [6]. VEST (Vascular Graft Solutions, Tel Aviv, Israel) is a cobalt chrome kink-resistant braid that stabilizes the SVG post implantation without changing CABG technique. Since 2011, this external stenting technology has been investigated by multiple international groups which evaluated its biological effects on disease progression through imaging-based metrics. These randomized trials, in which randomization was performed at the graft level, yielded consistent evidence about the VEST biological mechanism of action demonstrating significant mitigation of intimal hyperplasia (IH) and lumen irregularities by improving flow patterns and reducing SVG wall tension [7–11].

However, notable limitations of these studies and their design were the strictly standardized CABG technique (isolated CABG, on pump, minimum of 2 SVG etc.), the placement of only 1 VEST per patient, and the relatively short-term patient follow up.

The main objective of this multicentric study was to enhance generalizability of the focused randomized controlled trials (RCTs) by evaluating the short- to mid-term clinical outcomes, focusing on the need for revascularization in territories treated with VEST supported versus unsupported SVG, in a real world, heterogeneous CABG practice.

## Methods

Three centers independently enrolled a prospective registry of patients undergoing VEST-enhanced CABG, collecting baseline and perioperative data as well as long-term clinical outcomes. The study was approved by the local ethics committees and all patients gave informed consent (NCT06500897).

In each center, expert coronary surgeons trained in VEST use performed all CABG surgeries, with or without concomitant procedures, at least one SVG supported with an external stent (VEST, Vascular Graft Solutions, Israel), either on or off pump, and additional arterial and/or venous grafts as clinically indicated.

Open or endoscopic vein harvesting techniques were used and vein preservation was performed according to the routine practice of each center. A specific VEST model was chosen based on SVG diameter and length to create a stable SVG-VEST graft. In case of sequential grafts, either all vein segments or only the proximal part were VEST supported according to surgeon preference. After CABG, patients were prescribed statins, anti-platelets, and beta blockers according to practice guidelines. Selection of target graft for VEST support, in case of not fully VESTed multiple venous conduits and/or anastomoses, was left to the operator judgement.

No modifications of standard routine practice were introduced, preserving well established excellence pathways based on surgeon and center preference.

In-hospital adverse events were recorded including death, myocardial infarction (MI), stroke, transient ischemic attack, need for repeat revascularization and associated target vessels. All patients were systematically followed for Major Adverse Myocardial and Cerebral Effects (MACCE) via on site visits or phone interviews. In case of revascularization, granular information was obtained regarding the treatment method and the specific revascularized coronary vessel (VEST SVG/non-VEST SVG/arterial graft/De-novo target territory).

The primary endpoint was defined as incidence of repeat revascularization, evaluated as a whole and stratified for type of territory (VEST SVG/non-VEST SVG/arterial graft).

Secondary endpoints were major adverse cardiac and cerebral adverse events (MACCE). Continuous variables were tested for normality then expressed as mean and standard deviation or median and interquartile range as appropriate. Discrete variables were expressed as direct incidence and percentage. Either T-Test or Mann Whitney Test were used to test correlation of continuous variable while X square or Fisher Exact Test were used to treat categorical variable correlation hypothesis, which applies to the primary endpoint. Freedom from event graphs were plotted according to Kaplan Meier technique, while Cox regression was used to test association between time dependent variables and outcomes. Archiving was performed first on dedicated Microsoft Excel Datasheets then imported in IBM SPSS 22.0 for statistical processing.

## Results

A total of 397 patients who underwent CABG between September 2015 and December 2023 at the hands of 12 surgeons in 3 international sites, and were implanted with one or more external stents, were included in the study. Baseline patient characteristics are presented in Table 1. The mean (SD) age was 65 (8.63) years. Common cardiovascular risk factors were hypertension (91.7%), dyslipidemia (70.8%), and diabetes (40.1%). None of the patients had previous cardiac surgery.

**Table 1:** Baseline and pre-operative characteristics

	Mean ± SD or n/N (%)
**Age (yrs.)**	65.1 ± 8.63
**Gender (male)**	339/397 (85.39%)
**BMI**	27.2 ± 4.2
**Hypertension**	364/397 (91.69%)
**Hyperlipidemia**	281/397 (70.78%)
**Diabetes**	159/397 (40.05%)
**Smoking status**	
** Never smoked** ** Current/Ex smoker** ** Unknown**	154/397 (38.79%) 195/397 (49.12%) 48/397 (12.09%)
**Prior stroke**	5/397 (1.25%)
**Ejection fraction ≤ 50%**	127/397 (31.99%)
**Euroscore II**	1.26 ± 1.18

BMI: body mass index

Intra- and peri-operative data are reported in Table 2. The majority (73.3%) of patients underwent on-pump CABG, 6.8% of patients had concomitant valve surgery and the average number of grafts per patient was 3, more than half of which were saphenous vein grafts. Overall, 469 external stents were implanted, accounting for 71.7% of all 654 vein grafts. In 61.7% of patients (*n* = 245) all vein grafts were externally stented, whereas in a minority of patients (38.3%. *n* = 152) only 41.6% of SVGs were supported, Table 3. SVGs were stented and non-stented in similar proportions in both left (54.6% vs. 53.0%) and right (45.4% vs. 47.0%) coronary territories, respectively.

**Table 2:** Surgery data

	Mean ± SD or n/N (%)
**Isolated CABG** ** Concomitant valve surgery** ** Other concomitant procedure**	347/397 (87.40%) 27/397 (6.80%) 23/397 (5.79%)
**SVG Harvesting:**	
** Open** ** Endoscopic** ** Other/UNK**	319/397 (80.35%) 73/397 (18.39%) 5/397 (1.26%)
**Off pump**	106/397 (26.70%)
**Total number of grafts per patient** ** Number of arterial grafts per patient** ** Number of vein grafts per patient**	3.05 ± 0.85 1.40 ± 0.69 1.66 ± 0.68
**Total number of vein grafts** ** VESTed** ** Non-VESTed**	654 469/654 (71.71%) 185/654 (28.3%)
**Patients with all vein grafts VESTed**	245/397 (61.71%)
**Total number of arterial grafts**	568
**Arterial graft to LAD**	376 (94.71%)
**BIMA**	151 (38.03%)
**Sequential grafts**	136 (34.26%)

CABG: Coronary artery bypass grafting, SVG: Saphenous vein graft, LAD: left anterior descending, BIMA: bilateral interior mammary artery, UNK: Unknown

**Table 3:** Surgical attributes and revascularization rates by completeness of VEST placement

	Patients with all SVG VESTed (N = 245)	Patients with partial SVG VESTed (N = 152)
**Number of grafts per patient** ** Number of arterial grafts per patient** ** Number of SVG per patient**	3.5 1.3 2.3	2.8 1.5 1.3
**Total number of SVG** ** Left territory targets** ** Right territory targets**	311 151/311 (48.55%) 160/311 (51.45%)	343 203/343 (59.18%) 140/343 (40.82%)
**Patients with any revascularization procedure**	13/245 (5.3%)	10/152 (6.6%)

SVG: Saphenous vein graft

No technical failure in external stent deployment was reported.

In-hospital mortality occurred in two cases (0.5%) and post-discharge clinical follow up was completed for 97.2% of patients (*n* = 386) with a median (range) duration of 24 (1–101) months.

**Fig. 1:**
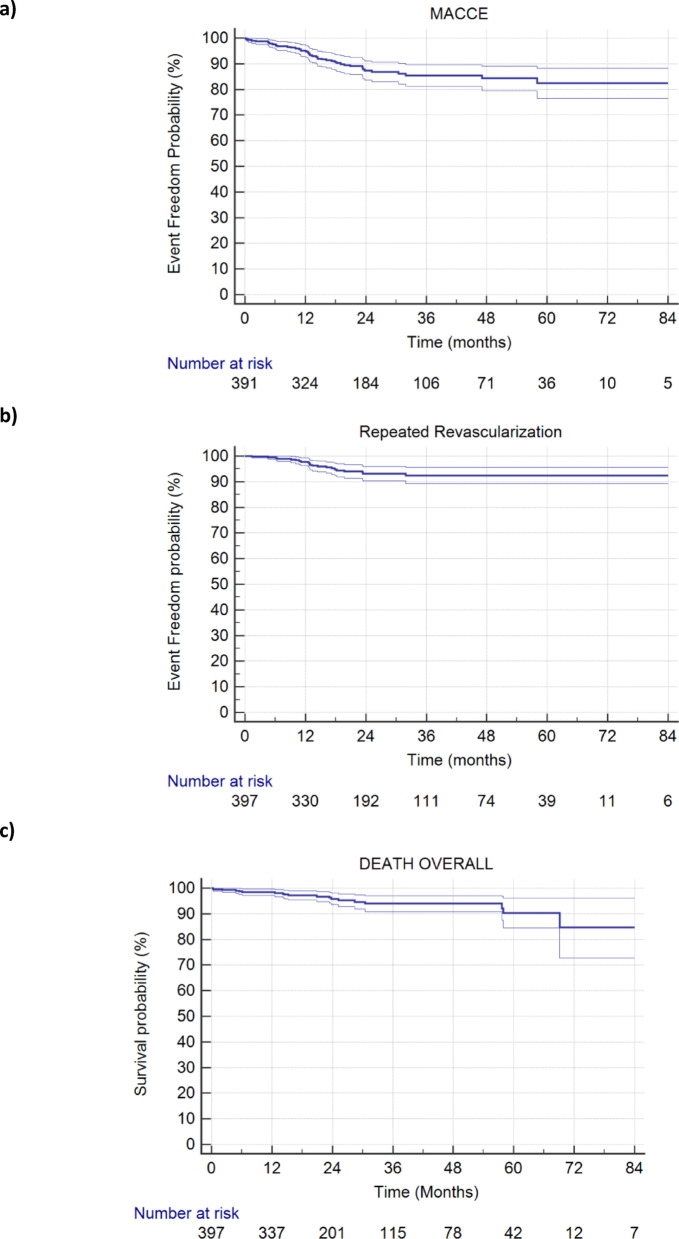
Freedom from clinical outcomes: **a**) MACCE; **b**) Repeat revascularization; **c**) Death

Freedom from MACCE at 1, 3, and 5 years was 95.1% (SE 0.011), 85.4% (SE 0.021), and 82.4% (SE 0.030) respectively (Fig. 1a). Freedom from repeat revascularization at 1, 3, and 5 years was 97.8% (SE 0.007), 92.4% (SE 0.016), and 92.4% (SE 0.016) respectively (Fig. 1b). Overall survival at 1, 3 and 5 years was 98.4% (SE 0.006), 93.9% (SE 0.015), and 90.3% (SE 0.029) respectively (Fig. 1c).

**Fig. 2:**
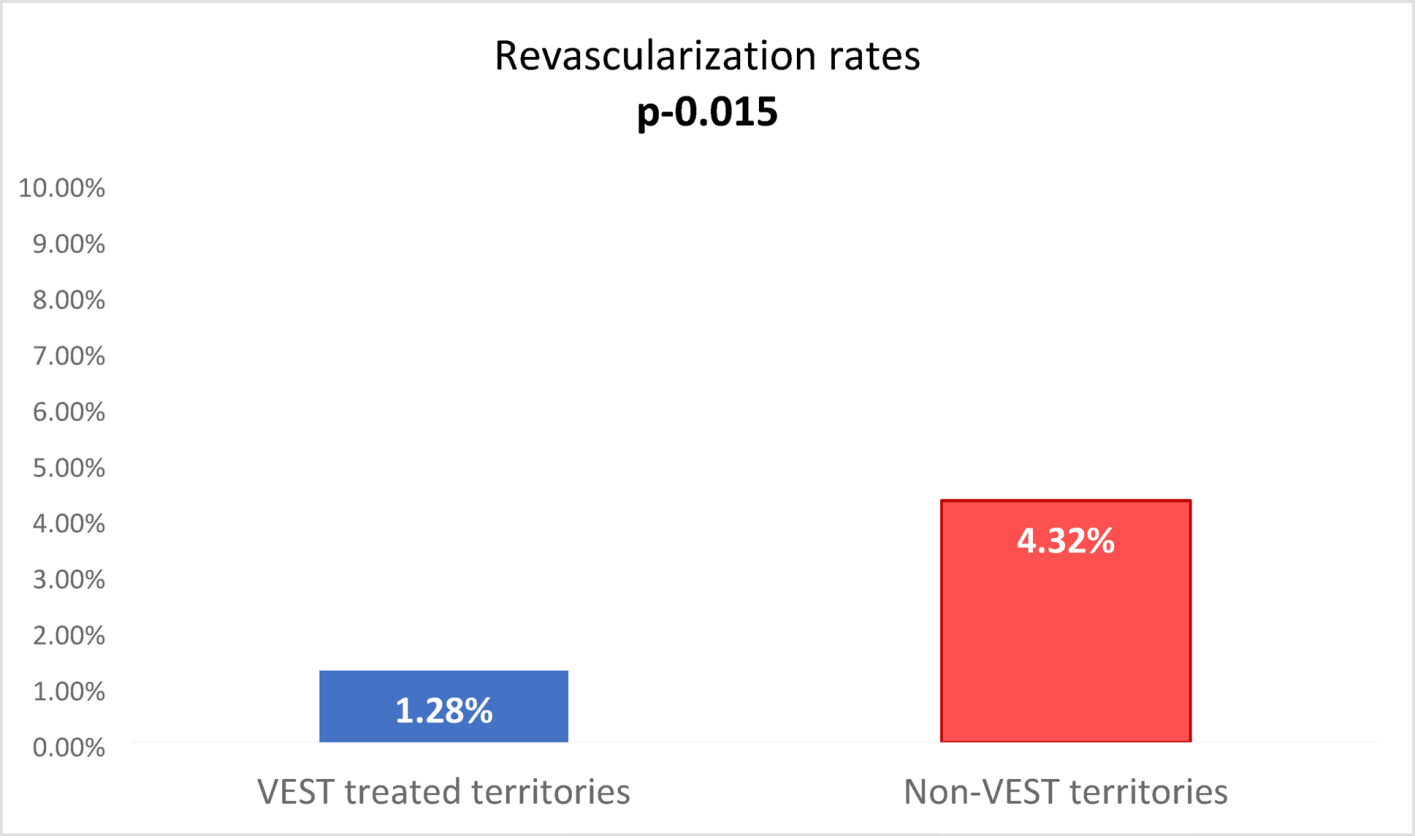
Graft level revascularization rates in previously saphenous vein grafted territories with/without external stents

Revascularization rates were 5/568 (0.90%) in territories grafted with arterial conduits, 6/469 (1.28%) in territories with stented SVGs, and 8/185 (4.32%) in territories grafted with non stented SVGs. The difference in revascularization rates between supported and unsupported veins proved to be statistically significant (*p* = 0.015, Fig. 2), while the difference between arterial and stented SVG territories was not significant (*p* = 0.61). On the patient level, revascularization rates were higher for 152 patients where not all vein grafts were supported by VEST compared to the majority 245 where all SVGs were supported with 6.6% and 5.3% respectively. It should be noted that the former group on average received more grafts per patient (3.5) than the latter group (2.8), possibly suggesting more severe coronary artery disease and a higher risk for revascularization.

## Discussion

This study is the largest report evaluating the short- to mid-term clinical outcome of VEST-enhanced CABG. The relatively large cohort encompasses a real world scenario with all kinds of targets, conduits and operational variants.

Our evidence suggests that VEST-enhanced CABG is feasible, safe and associated with low MACCE over several years post CABG.

In terms of feasibility, safety, and efficacy at the early stages, our findings are in line with Dushaj et al. [12] recently published peri-operative data for a cohort of 150 CABG patients with 205 externally stented vein grafts and 207 patients with 337 non-stented vein grafts. Significantly higher graft level SVG patency rates at discharge were reported for the VESTed vein grafts (93.8% vs. 87.8%, *p* = 0.05) resulting in a reduced occurrence of perioperative vein graft percutaneous coronary intervention. Kinking was observed as the cause of occlusion in 20% of bare vein grafts and was not observed in any of the externally stented SVGs. These early signs of potential clinical benefit of external stents are in line with other reports on lower rates of ischemic driven revascularization in externally stented compared to non-stented SVG at 2−4.5 years [8, 10], however the number of overall events is too small to draw robust conclusions.

Between 2011 and 2021, the safety and mechanism of action of external SVG stenting was evaluated in multiple within-patient randomized, controlled trials (RCTs) [8–11]. Over follow up periods of 1–4.5 years, external SVG stenting has shown progressive reduction of SVG IH by 12–14% and 22% at 1- and 2-years post CABG respectively [8–11]. A similar progressive effect was also angiographically demonstrated with significant reduction in lumen irregularities [13] at 2- and 4.5-years post CABG [8, 10]. The RCTs demonstrated, at the graft level, that VEST mitigates vein graft disease, while the current study demonstrates that VEST beneficial effect is maintained when broadening the patient population and surgical techniques and translates into an improvement in clinical outcomes, specifically the need for additional target vessel revascularization procedures. These observations strengthen pervious reports [13, 14] on the relationship between intimal hyperplasia, lumen irregularities and clinical outcomes.

At the patient level composite MACCE rates at 1, 3, and 5 years were 4.9%, 14.6%, and 17.6%, respectively (Fig. 1a). To put these numbers in perspective, they are compared to two large multicenter CABG studies with similar cohorts (Table 4). The SYNTAX trial reported considerably higher MACCE rates for 1251 European CABG patients at 1, 3, and 5 years post CABG of 10%, 17%, and 24% respectively [15]. The more recent data from the Duragraft registry [16] also reported higher MACCE rates of 7.6% at one year.

**Table 4:** Comparison of patient and surgical characteristics to published historical datasets

	VEST (N = 397)	SYNTAX EU CABG [15] (n = 1251)	Duragraft registry [16] (N = 2532)
**Age (yrs.)**	65.1 ± 8.63	65.3 ± 9.4	67.4 ± 9.2
**Gender (male)**	85.4%	82%	82.5%
**BMI**	27.2 ± 4.2	27 IQR 25–32	28.5 ± 4.5
**Hypertension**	91.7%	72%	84.6%
**Hyperlipidemia**	70.8%	76%	77%
**Diabetes**	40.05%	33%	44.6%
**History of Smoking**	49.12% 12% Unknown	66%	61.9%
**Prior stroke**	1.25%	5%	Not reported
**Ejection fraction**	32.0% ≤ 50%	23% ≤ 50%	32.1% ≤ 50%
**Euroscore II**	1.26 ± 1.18	Not reported	1.4 (median)
**Off pump surgery**	26.7%	13%	17.3%
**Concomitant procedure**	12.6%	0%	0%
**Vein harvesting technique**			
** Open** ** Endoscopic** ** Other**	80.4% 18.4% 1.26%	Not reported	80.6% 14.7% 4.6%
**Total grafts per patient**	3.05 ± 0.85	3 IQR 2–3	2.7 ± 0.8
**Vein grafts per patient**	1.66 ± 0.68	1 IQR 1–2	1.5 ± 0.9
**Arterial grafts per patient**	1.40 ± 0.69	1 IQR 1–2	1.2 ± 0.6

Revascularization rates at 1, 3, and 5 years were 2.2%, 7.6%, and 7.6% respectively (Fig. 1b). In comparison, the SYNTAX trial reported for their European CABG cohort similar revascularization rates at 1, 3, and 5 years post CABG of 3.7%, 7.5%, and 10% respectively [15]. The more recent data with a contemporary endothelial damage inhibitor [16] reported similar revascularization rates of 2.2% at one year.

Of course, at the patient level, this is not “per se” a demonstration of correlation between VEST use and clinical outcomes as these good results might be due to the excellence of the recruiting centers. However, for revascularization at the graft level it is interesting to observe a radical difference between VEST enhanced vein grafts and unsupported vein grafts in the hands of the same surgeons: target vessel revascularization rates were over 3 times higher in the unsupported territories as compared to externally stented SVG territories (Fig. 2). These findings may be explained by the inhibition of vein graft disease and enhancement of lumen uniformity as previously demonstrated and associated with VEST [8–11], as well as the minimization of peri-operative vein graft occlusion due to enhanced kink-resistance of VEST [12]. These findings in a heterogenous group of real-world patients that underwent off and on pump CABG, with and without concomitant procedures, with and without sequential grafting, using open and endoscopic saphenous vein harvesting, provide a first insight into the clinical outcomes of external stenting at the graft and patient levels.

A notable limitation of this study is potential surgeon selection bias. In a majority of patients no such selection was necessary, as all vein grafts were supported by VEST. An equal distribution of supported and unsupported grafts to the right and left territories confirms equality of target selection decisions. However, no post-hoc statistical technique can nullify the potentially hidden selection bias of surgeons systematically supporting the better vessel, as no systematic data of conduit and target quality and diameter are available in the dataset. Hence, this remains a potential limitation of the study, but the cohort is large enough and the heterogeneity of multiple surgeons in different centers performing the operations allows at least some degree of hidden selection bias attenuation.

While randomized controlled trials (RCTs) minimize selection bias and enable evaluation of treatment effect, their results usually apply to a specific sub population and do not necessarily reflect real world practice, which is more complex and diversified. Therefore, properly conducted real world studies have an important role in providing insights into the risk-benefit profile of a new intervention and in enhancing the generalizability of observations from RCTs. To quote Lee et al. [17], “The tension between the narrow focus and lack of generalizability of traditional RCTs and the inherent biases of observational analyses has led to confusion when results appear to differ.” The observations from this current study are in line with the imaging based VEST data previously generated by RCTs [8–11] and therefore are a source of confidence and not of confusion.

Lack of a control arm for patient level clinical outcomes in which none of the SVG were externally stented is another limitation of our study. Such a control arm would have provided a more definitive answer regarding the clinical effectiveness of VEST. Another limitation is the inconsistent rate of enrollment over the years, with a gradual increase as the learning curve proceeded, which explains the relatively low number of patients in the first segment of time.

## Conclusions

VEST-enhanced CABG is safe and feasible in real world routine practice which includes on and off pump CABG, sequential grafting, and concomitant valve surgery. Short- to mid-term clinical follow up suggests that VEST enhanced CABG is associated with low revascularization rates, and these interventional procedures are mostly to non-VESTed SVG territories.

## Data Availability

All relevant data within this article will be shared upon reasonable request to the corresponding author.
